# Antitumor activity of novel pyrazole-based small molecular inhibitors of the STAT3 pathway in patient derived high grade glioma cells

**DOI:** 10.1371/journal.pone.0220569

**Published:** 2019-07-30

**Authors:** Liang Zhang, Timothy E. Peterson, Victor M. Lu, Ian F. Parney, David J. Daniels

**Affiliations:** Department of Neurosurgery, Mayo Clinic, Rochester, MN, United States of America; Sechenov First Medical University, RUSSIAN FEDERATION

## Abstract

Abnormal activation of signal transducer and activator of transcription 3 (STAT3) transcription factor has been observed in many human cancers with roles in tumor initiation, progression, drug resistance, angiogenesis and immunosuppression. STAT3 is constitutively activated in a variety of cancers including adult high grade gliomas (aHGGs) such as glioblastoma (GBM), and pediatric high grade gliomas (pHGG). Inhibiting STAT3 is a promising target-specific chemotherapeutic strategy for tumors with aberrant STAT3 signaling. Here we investigated the antitumor effects of novel pyrazole-based STAT3 pathway inhibitors named MNS1 (Mayo Neurosurgery 1) in both pediatric and adult HGG tumor cells. MNS1 compounds selectively decreased cell viability and proliferation in patient-derived HGG cells with minimal toxicity on normal human astrocytes. These inhibitors selectively blocked IL-6-induced STAT3 phosphorylation and nuclear localization of pSTAT3 without affecting other signaling molecules including Akt, STAT1, JAK2 or ERK1/2 phosphorylation. Functional analysis showed that MNS1 compounds induced apoptosis and decrease tumor migration. The anti-tumor effects extended into a murine pHGG (diffuse intrinsic pontine glioma) patient derived xenograft, and systemic toxicity was not evident during dose escalation in mice. These results support further development of STAT3 inhibitors for both pediatric and adult HGG.

## Introduction

**Fig 1:**
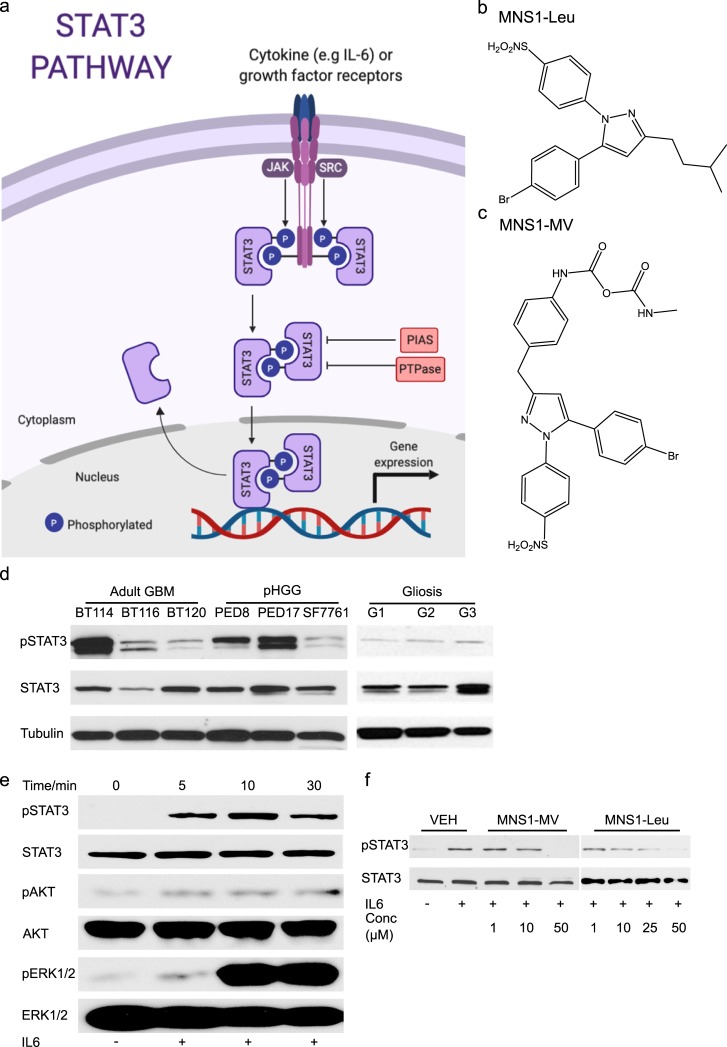
Pyrazole-based MNS-1 inhibitors block STAT3 phosphorylation. (a) Diagram of STAT3 pathway; activation is by cytokines, most commonly IL-6, and growth factor receptors, most commonly gp130. Phosphorylated STAT3 forms a reciprocal homodimer that translocates into the nucleus where it interacts with DNA and activates gene transcription. The activated form is typically inhibited by Protein inhibitor of activated STAT (PIAS) and Protein-tyrosine Phosphatase (PTPase). (b) Chemical structure of MNS1-Leu. (c) Chemical structure of MNS1-MV. (d) Western blot of pSTAT3 (Y705) in adult GBM and pHGG patient derived cell lines at baseline compared to non-tumor brain control (removed for epilepsy). (e) Western blot analysis of IL-6 (100 ng/mL) stimulation of STAT3 phosphorylation in dBT114 for 5–30 min. (f) Western blot analysis on the effect of IL-6-induced STAT3 phosphorylation in dBT114 in presence of MNS1-MV and MNS-Leu in various concentrations after a 10 min exposure to IL-6 stimulation.

The signal transducer and activator of transcription (STAT) proteins are a family of transcriptional factors that are activated in response to growth factors and cytokines and promote cell proliferation and survival [1]. Canonical STAT3 activation works through recruitment of STAT proteins occurs through the Src homology 2 (SH2) domain to receptor phospho-tyrosine motifs which promotes phosphorylation and activation of a critical tyrosine residue in the SH2 domain of STAT proteins by Janus kinases (JAKs) and Src kinase families via cytokines such as interleukin-6 (IL-6) [2, 3]. Phosphorylation facilitates STAT-STAT homodimerization, leads to nuclear translocation and promotes gene transcription by binding specific DNA-response elements in the promoters of target genes promoting proliferation and survival (**Fig 1A**) [4]. In normal cells, the activation of STAT proteins is very transient and strictly regulated. However, convincing evidence has shown that STATs play a key role in oncogenesis [4, 5]. Specifically, STAT3 has been found to be aberrantly active in numerous cancers including leukemia, lymphoma, breast, lung and malignant brain tumors [6–8]. Inhibition of STAT3 signaling has been shown to inhibit cancer cell growth and induce apoptosis indicating STAT3 is a promising therapeutic target for cancer [7–10].

Adult high grade gliomas (aHGGs), including glioblastoma (GBM), and pediatric high grade gliomas (pHGG) are the most aggressive primary brain tumors treated in practice, and have a median survival of approximately 14 months despite optimal therapy (surgery, radiation, and chemotherapy) [11]. There is strong evidence that STAT3 is a key signaling molecule involved in tumor progression and STAT3 activation affects proliferation, growth, and apoptosis [6–8, 12]. A number of anti-cancer compounds that inhibit STAT3 have been designed to date including Stattic[13, 14], the first direct small molecular inhibitor, as well as LLL12 [9], S31-201[10] and STA-21[15], in addition to the dual STAT3/STAT5 inhibitor SH-4-54[16]. In a similar setting, upstream inhibitors of STAT3 signaling, predominately acting at JAK2, have also been developed including WP1066[7], and OPB-31121[17]. These inhibitors are in various experimental stages and initial clinical trials have been disappointing due to toxicity issues [17, 18]. The most progressed inhibitor is the upstream JAK2 inhibitor, WP1066, which is currently being investigated in a recently commenced Phase I study in recurrent aHGG [19].

Due to these reasons we turned our attention to develop new STAT3 inhibitors that are easy to synthesize, selective for malignant glioma cells and have potent anti-cancer agents. To this end, we developed a series of pyrazole compounds named MNS1 (Mayo Neurosurgery 1 series) which inhibit STAT3 phosphorylation and downregulate STAT3 downstream targets. Therefore, the primary objective of this study was to validate and assess feasibility of the anti-tumor properties of these MNS1 compounds in a series of patient-derived HGGs to provide the platform for further therapeutic advances in the management of these dismal diseases.

## Methods

### Cell lines

For all human tissue studies, informed consent was obtained after Institutional Review Board approval granted. Patient derived neurosphere cell lines MC-BT114, MC-BT116 and MC-BT120 were from surgical samples of adult HGG patients per our established protocol[20]. MC-PED8, MC-PED17 and SF7761 were derived from pHGG patients. The tumor tissues were harvested during surgery at the Mayo Clinic, collected in serum-free DMEM/F12, and transported to the laboratory on the same day (except SF7761 which was a gift from Dr. Hashizume) [21]. The tissue was spun and the pellet was broken into small pieces with trituration using a 1 mL pipette tip followed by pass through a 40 μm cell strainer before plated in media hormone mix serum-free complete media (MHM^+++^) which consisted of DMEM/F12 (Gibco), 25 mM glucose (Sigma), sodium bicarbonate (Gibco), 2 mM glutamine (Gibco), HEPES (Gibco), penicillin/streptomycin (Gibco), N2 supplement (Gibco), 4 μg/mL heparin (Sigma), 20 ng/mL human EGF (PeproTech), 20 ng/mL human b-FGF (PeproTech), 20 ng/mL human PDGF AA and PDGF BB (Shenandoah). Cells cultured as tumor neurospheres were passaged every 1–2 weeks when appropriate.

Differentiated, adherent MC-BT114 (dBT114), MC-BT116 (dBT116), MC-BT120 (dBT120) and MC-PED17 (dPED17) were tumors that were cultured in Dulbecco's Modified Eagle Medium/ Nutrient F12 (DMEM/F12, Sigma, St Louis, MO) media containing 10% fetal calf serum (FCS). Differentiated cells were used when performing assays with relatively short duration (<10 minutes) to maintain reliability of the results.

HEK293T embryonic kidney cells were purchased from American Type Culture Collection (ATCC, Manassas, VA) and were cultured in DMEM (Sigma) with 10% FCS. Primary normal human astrocytes (NHA) were obtained from Gibco (Grand Island, NY) and were cultured in DMEM media containing 10% FCS and 1X N2 supplement (Gibco). Fresh surgical cortical gliosis samples were obtained from separate non-tumor patients.

### Reagents

MNS1-Leu (molecular weight 448.38 g/mol) and MNS1-MV (molecular weight 612.15 g/mol) were synthesized and characterized at the Mayo Clinic (U.S. Patent 10,138,208 B2), the compounds were dissolved in dimethyl sulfoxide (DMSO, Sigma-Aldrich, St. Louis, MO, USA). Stock solutions were made at a concentration of 10 mM in DMSO and stored at -20°C.

### Cell viability assay

The cytotoxic effect of MNS1 series compounds was determined using CellTiter-Blue Cell Viability Assay (Promega, Fitchburg, WI), according to the manufacturer’s instructions. Cells (5x10^3^ cell/well, 100 μL) were plated in culture media in black well, clear bottom, 96-well microplates (Corning Costar, Cambridge, NY), and cultured overnight at 37°C, with 5% CO_2_. The next day, cells were treated with varying concentrations (0.05–25 μM) of MNS1 analog for 72 h. All drugs were dissolved in 100% dimethyl sulfoxide (DMSO, Sigma), and given to cells at a final concentration of 0.5% DMSO. The fluorescence read out was measured using an Infinite M200 Pro microplate reader (Tecan, Chapel Hill, NC). The viability of vehicle-treated (0.5% DMSO) cells was considered as 100%. The potency (50% inhibitory concentration, IC_50_) of each drug was calculated using Prism 6 (GraphPad Software, San Diego, CA). Cell viability assays were repeated for NHA cells, in lieu of the tumorigenic cell lines, to assess cancer-selective toxicity of the MNS1 analogs.

### IL-6 induction of STAT3 phosphorylation

For IL-6 stimulation, differentiated cells were seeded in culture media for 24 h, followed by serum depletion for 12 h. Cells were then treated with inhibitor or DMSO for 1 h, prior to stimulation with 100 ng/ml rhIL-6 (Pepro Tech Inc., Rocky Hill, NJ), for 0, 5, 10, or 30 min. This dose and time course have been reported previously for cancer studies of IL-6.[22–25] Further time points would have been considered if changes in the markers of interest were not observed. Cells were then flash frozen in an ETOH/dry ice bath and analyzed by Western blot analysis.

### Luciferase assays

Lentiviral particles containing a STAT3-responsive firefly luciferase construct reporter gene (SABiosciences, Valencia, CA) were transduced into HEK293T cells, according to the manufacturer’s instructions, and selected by 2 μg/mL puromycin (Sigma-Aldrich), for one week, to establish a stable cell line. Cells (3x10^5^ cells/well) were then plated into 96-well microplates (Corning Costar, Cambridge, NY) in complete culture media, which was then depleted of serum for 12 h, prior to drug treatment for 3 h followed by IL-6 stimulation for another 3 h. After drug treatment and IL-6 stimulation, the cells were washed once with ice-cold Dulbecco’s phosphate-buffered saline (DPBS, Gibco, Grand Island, NY) before lysed in 20 μL 1x reporter lysis buffer (Promega, Fitchburg, WI). Luminescence values were then determined using a Luciferase Assay System (Promega), according to the manufacturer’s instructions, using Infinite M200 Pro microplate reader (Tecan). The relative luciferase units (RLU) of vehicle-treated (0.5% DMSO) cells were considered as 100%. The STAT3 activation of each drug was calculated and plotted using Prism 6 (GraphPad Software, San Diego, CA).

### Preparation of nuclear extract

Cells were plated in culture media in 10-cm plates at densities sufficient to reach 90% confluency within 1 day, followed by 12 h serum depletion. The cells were then treated with either vehicle (0.5% DMSO) or MNS1 compounds for 1 h prior to IL-6 stimulation and harvested. A previous protocol for nuclei extraction was adopted with modification [26]. Briefly, adherent cells were set on ice, rinsed once with 5 mL ice-cold DPBS, once with 5 mL ice-cold hypotonic buffer (20 mM HEPES pH 7.9, 1 mM EDTA, 1 mM EGTA, 20 mM NaF, 1 mM Na_3_VO_4_, 1 mM Na_4_P_2_O_7_, 1 mM DTT, and 1x Complete Protease Inhibitor (PI) Cocktail, Boehringer Mannheim, Indianapolis, IN), and scraped into 300 μL hypotonic buffer containing 0.2% Nonidet P-40 and 1x PI. The cell lysates were then incubated on ice for 10 min, before pelleting at 15,000 rpm for 20 s. The supernatant, representing the cytoplasmic fraction, was then stored at -70°C until further use. The nuclei pellets were resuspended in 50 μL high salt buffer (420 mM NaCl, 20 mM HEPES pH 7.9, 1 mM EDTA, 1 mM EGTA, 20% glycerol, 20 mM NaF, 1 mM Na_3_VO_4_, 1 mM Na_4_P_2_O_7_, 1 mM DTT, and 1x PI), incubated at 4°C for 45 min on a rotating wheel, and cellular debris pelleted by centrifugation at 12,000 rpm, for 20 min, at 4°C. Supernatants were then transferred to fresh tubes, in small aliquots, and stored at -70°C. Protein concentrations were determined by Pierce BCA protein assay (Thermo Fisher Scientific, Rockford, IL), according to the manufacturer’s instructions, prior to electrophoresis.

### Western blot analysis

Cells were washed twice in ice-cold PBS, and collected in lysis buffer (10 mM HEPES pH 7.4, 50 mM NaCl, 50 mM NaF, 50 mM sodium pyrophosphate, 5 mM EDTA, 5 mM EGTA, 2 mM Na_3_VO_4_, 1% Triton X-100, 0.5 mM PMSF, and 10 mg/mL leupeptin), followed by scraping and 5 s sonication, to achieve a homogeneous solution. Protein concentrations were then determined by Bradford assay (Bio-Rad; Hercules, CA). To characterize changes in protein levels in whole cell lysates, 10 μg of total protein was size fractioned by 12% SDS-PAGE, transferred to PVDF membranes, and stained with Ponceau-S (Sigma-Aldrich), to ensure equal protein loading. For Western blot analysis, membranes were incubated for 1 h in blocking solution containing 1% non-fat dry milk in PBST (PBS and 0.5% Tween 20), followed by overnight incubation with one of the following primary antibodies purchased from Cell Signaling (Danvers, MA) against: STAT3 (124H6), pSTAT3 (Y705), pSTAT1 (Y701), STAT1, pJak-2 (Y1008), Jak-2 (D2E12),p-p44/42 MAPK (T202/Y204), p44/42 MAPK (Erk1/1), pAkt (S473) and Akt. Equal protein loadings were also determined by detection of α-tubulin (Sigma-Aldrich). Antibody-bound membranes were then incubated for 1 h with horseradish peroxidase-conjugated anti-IgG antibodies, and visualized using Super Signal West Pico enhanced chemiluminescence detection (Thermo Scientific, Waltham, MA).

### Apoptosis assays

Apoptosis was analyzed using an Annexin V apoptosis detection kit (Calbiochem, Rockland, MA). dBT114 (5x10^3^ cells/well) were plated in 6-well tissue culture plates, and allowed to reach 60% confluency, prior to the addition of MNS1 analogs for 24 h. Floating and adherent cells were then harvested and stained with FITC-labeled Annexin V and propidium iodide for 15 min at room temperature in the dark, washed with cold binding buffer, and immediately analyzed by flow cytometry for both Annexin V and propidium iodide.

#### Cell migration assays

Chemotaxis assays were performed using 8-μm pore Transwell plates (Corning Costar, Cambridge, NY). dBT116 cells (3x10^5^ cells/plate) were seeded in 60-mm plates at densities sufficient to reach 90% confluency within 1 day, followed by depletion of serum and treated with varying doses of MNS-1 analogs for 12 h. Following treatment, adherent cells were trypsinized, washed in DPBS, and live cells (1x10^4^ cells/transwell) then plated into the top chambers of the transwells in serum-free media. Culture media, containing 10% FCS, was added to the bottom well, to serve as a chemoattractant, and the plates incubated, at 37°C, under 5% CO_2_, for 16 h. Migrated cells (membrane underside) were then fixed, stained using Hema 3 (ThermoFisher), counted in at least 8 randomly selected high-power fields (20X) for each condition, and then photographed (Axiostar, Zeiss).

### Plasma stability

Samples were analyzed using LC-ESI-MS. The samples (25 μM) were incubated in human plasma at 37°C for 96 h. Aliquots (100 μL) were taken at time points 0, 3, 6, 12, 24, 48, and 96 h, and frozen for later analysis. Analysis began by allowing the samples to warm-up to room temperature. Ethyl acetate (800 μL) and sodium acetate (100 μL, 0.1M solution) were added to the samples, which were then mixed for 15 min. Then, 500 μL of ethyl acetate was removed and dried down over nitrogen gas and reconstituted in 100 μL of 1:1 DMSO:Ethyl Acetate and 400 μL of water. This solution was then injected directly into the LC-MS unit for analysis. Standard calibration curves for each compound were made using concentrations ranging from 0.1–100 μM and plasma stability was calculated relative to the concentration at time point 0 h.

### Establishment of cell-derived orthotopic DIPG xenografts

Female Hsd:athymic Nude Foxn1nu mice (20 g, Envigo) were kept under specific pathogen-free conditions in air-filtered cages and received food and water ad libitum. The mice were handled in accordance with IACUC guidelines and approval from Mayo Clinic Institutional Committee for Animal Research. The flank xenograft model was established in-house [27]. Animals were anesthetized with isoflurane and the 5×10^6^ cells/ 200 μL of SU-DIPG XVII cell/Matrigel (1:1, Corning Incorporated, Corning, NY) mixture was implanted subcutaneously into the right flank of the animals. The animals were observed two to three times per week for tumor development, tumor volume (LxWxH) was evaluated twice per week with a digital caliper. When the tumors reached approximately 100 mm^3^ by caliper measurement, the animals were randomized into vehicle and treatment groups (n = 5–10 per group) and received MNS1-Leu or vehicle once daily for consecutive of 21 days.

### *In vivo* dose escalation study, toxicity studies and bioluminescence imaging

Tumor bearing animals were treated with dose escalation (MNS1-Leu) with increment of 10 mg/kg per week starting from 10 mg/kg for 7 days to 30 mg/kg for 7 days. Animals were fasted for 4–6 h prior to oral gavage. The drug was dissolved in 10% DMSO followed by mixing with 90% PEG 300. Delivered at 10 μL/g of body weight. No animal exhibited physical or neurological deficits throughout the duration of drug treatment. Flank tumors were collected after 21 days of treatment and fixed in 4% PFA.

Animals were injected intraperitoneally with 10 mg/kg CycLuc1 (Millipore) solution, bioluminescence imaging was acquired 10 min after CycLuc administration using the IVIS-2000 Imaging System (Xenogen Corporation) and analyzed by LivingImage 4.3 software. Signal intensities were quantified within regions of interest, as defined by the LivingImage software.

### Immunohistochemistry

All images of stained slides were taken under same magnification (40X) and exposure time using EVOS FLc Imaging System (Thermo Fisher Scientific, Waltham, MA).

### STAT3 TR-FRET assay

For binding analyses, GST-STAT3 protein was purchased from SignalChem Corporate (Richmond, BC, CANADA). Tb-anti-GST and 1 M DTT were obtained from LifeTechnologies (Carlsbad, CA, USA). Fluorescent STAT3 peptide (5-FAM–G(pY) LPQTVCONH2) was made in house at Mayo Clinic using published methods.[14] STAT3 S3I-1757 was purchased from Glixx Laboratories (Southborough, MA, USA). 384-well black low volume plates were purchased from Corning Inc. (Corning, NY, USA). Sodium chloride, HEPES, EDTA, Triton-X100 were purchased from Sigma (St. Louis, MO, USA). Assay condition for IC_50_ determination under optimized condition: In 384-well black low volume plates with 20 μL/well final assay volume, fluorescent STAT3 peptide (10 nM) was incubated under room temperature for 20 min with 5 nM GST-hPXR-LBD and 5 nM Tb-anti-GST in assay buffer (50 mM NaCl, 10 mM HEPES, 1 mM EDTA, 0.01% Triton-X100, 2 mM DTT, pH 7.5) plus 0.2% methyl sulfoxide or Titrations of S3I-1757, MNS1-Leu, MNS1-MV, or STA-21 (100 μM to 370 nM with 1:2 dilutions for 9 concentration levels) in 0.2% DMSO. The TR-FRET emission signal (10,000 × 520 nm/490 nm) from each well was then collected using a PHERAstar FS plate reader from BMG Labtech (Durham, NC, USA) using a 340 nm excitation filter, 100 μs delay time, and 200 μs integration time. Raw data from the plate reader were directly used for analysis.

### Statistics

For comparison of two groups, data were analyzed using two-tailed Student t-test. For comparison of multiple groups, data were analyzed using one-way ANOVA. P<0.05 was considered statistically significant, and all analyses were conducted using GraphPad 8 (La Jolla, California).

### Ethics statement

All human tissue samples and analysis in this study were obtained under an approved ethics (#12–003458) by the Mayo Clinic Institutional Review Board. Written consent for tissue collection was obtained prospectively prior to surgery, with all analyses performed on anonymized samples. In the case of pediatric (minor) samples, written consent was obtained from the parents or guardians.

## Results

### MNS1 compounds inhibits IL-6-induced STAT3 phosphorylation in high grade gliomas

We synthesized novel pyrazole-based compounds and named the series MNS1 (Mayo Neurosurgery 1). Two of the pyrazole derivatives shown are MNS1-Leu (**Fig 1B**) and MNS1–MV (**Fig 1C**). Previously, our laboratory reported that there was a significant increase of multiple cytokines including interlukin-6 (IL-6) secreted in glioma-conditioned media from patient-derived adult GBM cell lines [28] which suggested these tumors have increased STAT3 signaling. Here, we first determined if the STAT3 pathway is activated at baseline in our HGG cell lines and then after IL-6 administration. We performed western blot analysis to confirm phosphorylated STAT3 Tyr705 (pSTAT3, Y705) was present in our cell lines; activated pSTAT3 was present at high basal level in both adult GBM and pHGG lines including dBT114 (adult GBM) and MC-PED8 (H3K27M pHGG) and MC-PED17 (H3K27M pHGG) cells (**Fig 1D**). Whereas, non-tumor brain controls (removed for epilepsy surgery), did not show appreciable activated STAT3. Using western blot analysis, IL-6 potently activated STAT3 phosphorylation in dBT114 cells with increased expression of pSTAT3 (Y705) in 5 min which lasted for 30 min **(Fig 1E**). There was also a delayed induction of pErk1/2 (T202/Y204) with no change observed in pAkt. To validate that our compounds inhibit STAT3 signaling, we looked at whether our compounds were capable of inhibiting IL-6-induced STAT3 phosphorylation *in vitro*. We found that MNS1-MV and MNS1-Leu potently inhibit STAT3 phosphorylation at 10 μM in dBT114 cells (**Fig 1F**).

### MNS1 compounds showed selective toxicity towards adult GBM and pediatric high grade glioma cells

**Fig 2:**
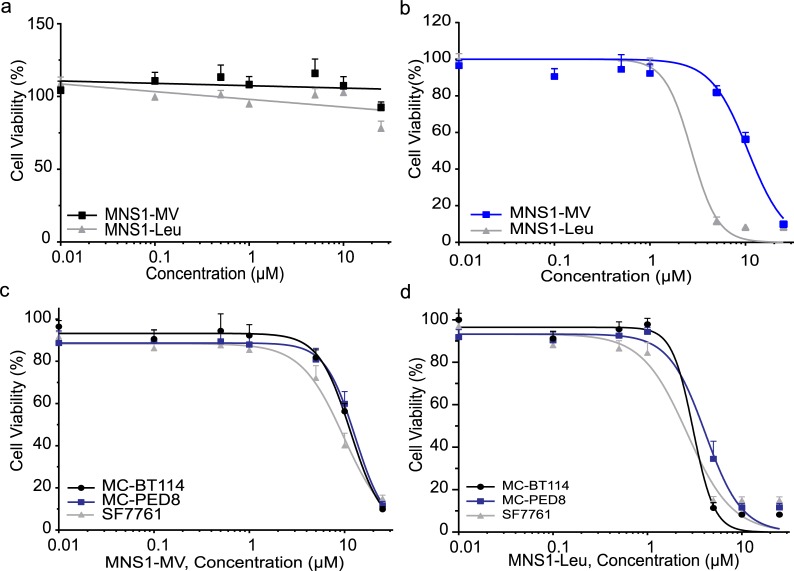
MNS1 compounds selectively inhibit the viability of adult GBM and pediatric glioma tumor cells. (a) Viability of control Normal Human Astrocytes treated with various concentrations of MNS1 compounds for 72 h show no toxicity up to 25 μM. (b) Dose-response curve of MNS1 analogs in MC-BT114. Dose-response curve in MC-BT114 (aGBM), MC-PED8 (H3K27M), SF7761 (H3K27M) tested with (c) MNS1-MV or (d) MNS1-Leu after 72 h treatment. Values are the means ± S.E.M (error bars) of triplicate experiments.

Selective toxicity is critical in cancer therapy; to determine whether the MNS1 compounds selectively killed cancer cells with minimal toxicity to normal brain cells, we tested the MNS1 compounds with treatments ranging from 0.01–25 μM. Indeed, treatment with MNS1-MV or MNS1-Leu showed none to minimal toxicity up to 25 μM for 72 h in normal human astrocytes (NHA) (**Fig 2A**). In contrast, the viability of GBM cell line MC-BT114 was inhibited by more than 90% in response to 25 μM of MNS1-MV or MNS1-Leu (**Fig 2B**). The IC_50_ values of MNS1-MV were 10.89 μM for MC-BT114 cells, 11.71 μM for MC-PED8 cells and 8.5 μM for SF7761 (**Fig 2C**). The IC_50_ values for MNS1-Leu were 2.99 μM for MC-BT114 cells, 3.83 μM for MC-PED8 cells and 2.42 μM for SF7761 (**Fig 2D**). Both adult GBM and pHGG showed similar sensitivity towards MNS1 compounds.

### MNS1 compounds inactivate STAT3 and inhibit its nuclear translocation

**Fig 3:**
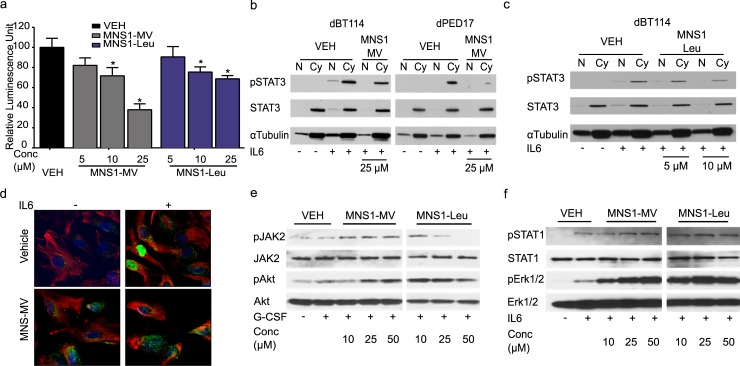
MNS1 compounds effectively inhibit STAT3 activation and nuclear translocation in both adult GBM and pediatric HGG cells. (a) Luciferase activity driven by a STAT3-responsive transcriptional element on an expression vector showed inhibition of STAT3 against IL-6 in presence of MNS1-MV or MNS1-Leu. Values are the means ± S.E.M (error bars) of triplicate experiments. One-way ANOVA was used to calculate statistics. * p<0.05. Subcellular fractionation analysis on STAT3 translocation into the nucleus upon IL-6 stimulation (100 ng/ml) in dBT114 or dPED17 in presence of (b) 25 μM of MNS1-MV or (c) 10 μM of MNS1-Leu. N denotes for nucleus fraction. Cy denotes for cytosol fraction. All experiments were repeated independently three times. (d) Confocal imaging of pSTAT3 in dBT114 in presence of MNS1-MV. Treatment of MNS1 analogs did not affect activation of (e) JAK2 and Akt upon G-CSF stimulation or (f) STAT1 and ERK1/2 activation upon IL-6 stimulation.

To gain insight into inhibition of the STAT3 pathway in HGG cells, we measured the transcriptional activity of STAT3 utilizing a STAT3-responsive luciferase assay transduced in HEK293T cells. Both MNS1-MV and MNS1-Leu significantly inhibit IL-6-induced STAT3 activation in a dose-dependent manner (**Fig 3A**). Once STAT3 is activated and phosphorylated, it translocates to the nucleus and functions as a transcription factor. Therefore, we analyzed the cellular localization of STAT3 in HGG cells after treatment with MNS1-MV and MNS1-Leu. In vehicle-treated cells (0.5% DMSO), both adult GBM and pHGG cells showed the absence of pSTAT3 in either nucleus or cytosol. Upon IL-6 stimulation, there is a drastic increase of pSTAT3 in the cytosol with translocation to the nucleus (**Fig 3B**). However, there is visible inhibition of IL-6-induced pSTAT3 translocation to the nucleus with loss of pSTAT3 expression at 25 μM of MNS1-MV in both dBT114 and dPED17 cells (**Fig 3B**). MNS1-Leu could inhibit IL-6-induced pSTAT3 translocation at a lower concentration of 10 μM in dBT114 cells, suggesting that MNS1-Leu is a more potent STAT3 pathway inhibitor in these lines (**Fig 3C**). These results were confirmed with confocal microscopy, where we found minimal pSTAT3 at baseline in dBT114 cells, but in the presence of IL-6 activated pSTAT3 was confined to the nucleus and this was blocked in the presence of MNS1–MV (**Fig 3D**). To evaluate whether our compounds interact with the phosphor-Y705–binding site in the SH2 domain of STAT3 we used a time resolved fluorescence resonance energy transfer (TR-FRET) assay.[29] Our compounds did not displace the fluorescent STAT3 peptide (5-FAM–G(pY) LPQTVCONH2) from STAT3, while control direct STAT3 inhibitor S3I-1757 did so at 62.1 ± 6.1 μM (**S1 Fig**). [14, 30]

STAT3 is activated in numerous ways including IL-6-induced JAK2 phosphorylation. To determine specificity and rule out JAK2 inhibition by MNS1 compounds, we examined the effect of our compounds on the phosphorylation of JAK2, STAT1, Erk1/2 and Akt. Western blot analysis showed that phosphorylated JAK2 was not affected by MNS1-MV and was significantly affected by MNS1-Leu only at very high concentrations in dBT114 cells (**Fig 3E**). Phosphorylated STAT1, Erk1/2 and Akt were not affected in any of the MNS1 compounds (**Fig 3F**). These results indicate that MNS1-MV and MNS1-Leu mostly inhibit STAT3 activation via IL-6-mediated STAT3 pathway and not direct JAK2 inhibition, except maybe at higher concentrations.

### MNS1 compounds induced apoptosis and inhibited migration

**Fig 4:**
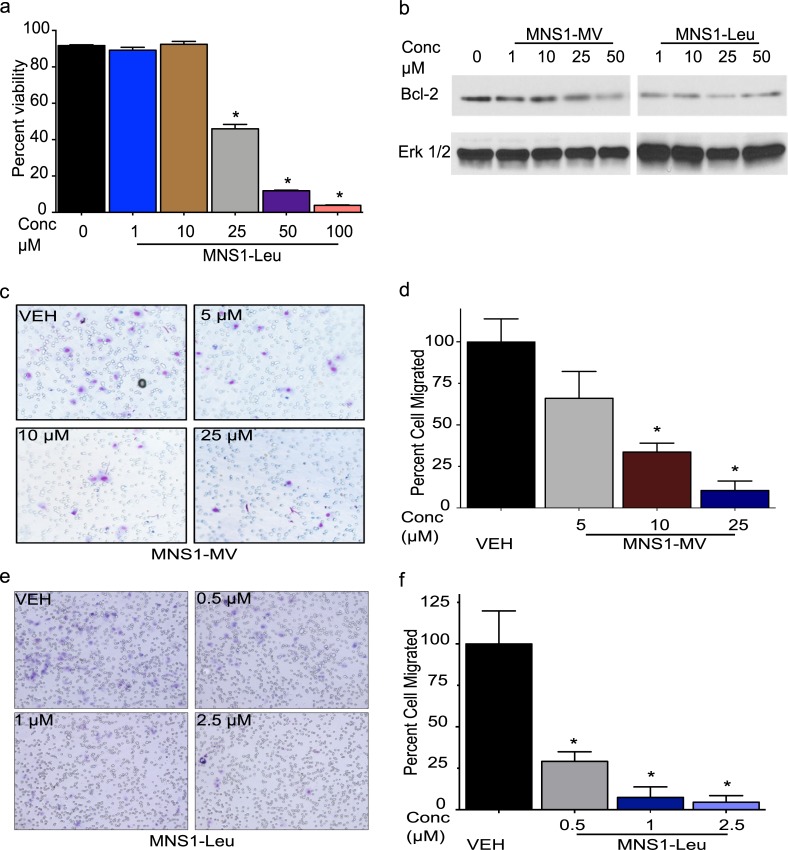
MNS1 compounds induce apoptosis and inhibit migration in adult GBM cells. (a) Flow cytometry analysis of annexin V/PI staining in dBT114 cells showed dose dependent decrease of cell viability, via apoptosis with treatment of MNS1-Leu for 24h. Values are the means ± S.E.M (error bars) of triplicate experiments. (b) Western blot analysis showed decreased expression of Bcl-2 with increased dose of MNS1-MV or MNS1-Leu. Total Erk1/2 was used as loading control. Images of (c) dBT116 (aHGG) migration assay and (d) quantification after MNS1-MV treatment. Images of (e) dBT116 migration assay and (f) quantification post MNS1-Leu treatment. Values are the means ± S.E.M (error bars) of triplicate experiments. 8 fields were imaged at 20X and counted in each experimental group.

Next, we investigated whether our compounds decreased HGG cell viability through induction of apoptosis. We performed flow cytometric analysis using Annexin V-FITC staining. Flow cytometric analysis of Annexin V/Propidium Iodine (PI) staining confirmed that MNS1-Leu regulates cell death through apoptosis in a dose dependent manner in dBT114 cells (**Fig 4A**). The IL-6/STAT3 pathway regulates a number of gene expressions such as Bcl-2 that mediate proliferation or inhibit apoptosis during tumor formation. We found that Bcl-2 expression was decreased with increased dose of MNS-Leu after 24 h exposure to the drugs in dBT114 (**Fig 4B**).

Migration and invasion are one reason for the high rate of recurrence and limited therapeutic response in malignant gliomas. We therefore used a migration assay to determine if MNS1 compounds could suppress cell migration *in vitro*. Adult GBM dBT116 cells showed significant reduction of migration post MNS1-MV or MNS1-Leu treatment. Treatment with MNS1-MV or MNS1-Leu significantly inhibited cell migration across a transwell membrane. The percent of cells migrated after MNS1-MV treatment were 33.6% at 10 μM and 10.5% at 25 μM after 12 h exposure (**Fig 4C and 4D**). The percent of cell migration after MNS1-Leu treatment was 29.1% at 0.5 μM, 7.4% at 1 μM at 4.5% at 2.5 μM after 12 h exposure (**Fig 4E and 4F**). These results collectively indicate that MNS1-MV and MNS1-Leu induce apoptosis, downregulate proliferation-related proteins and inhibit migration in the glioma cells.

### MNS1-Leu shows favorable *in vivo* effects with no toxicity and good stability in human plasma

**Fig 5:**
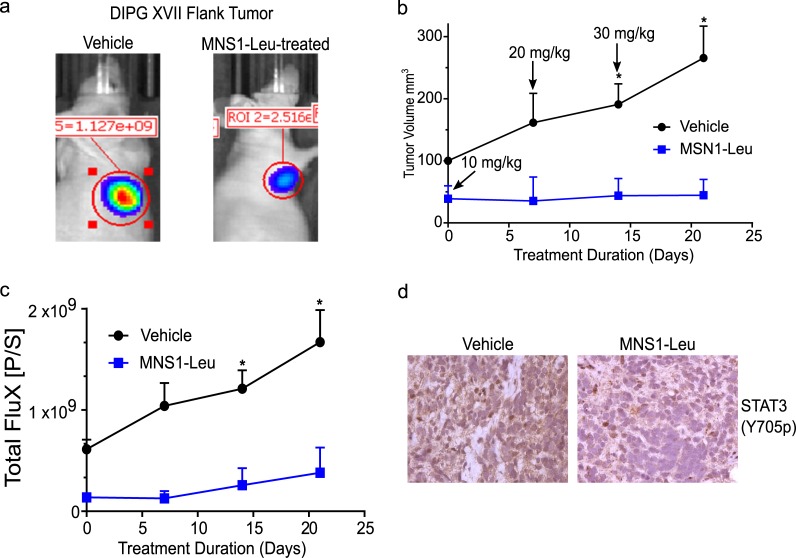
MNS1-Leu slows pHGG growth *in vivo* and reduces STAT3 activation. Dose escalation study with MNS1-Leu in PDX model of pHGG (DIPG17) starting at 10 mg/kg and increasing to 30 mg/kg over 21 days. (a) BLI imaging in PDX model of vehicle control vs. MNS1-Leu at day 21. (b) Caliper measurements of flank “DIPGXVII” xenograft tumors showed significant increase in tumor size when treated by vehicle, but not when treated by MNS1-Leu. (c) Similarly, bioluminescence of these tumors also showed significantly increased flux in those treated by vehicle, with no difference in those treated by MNS1-Leu. Values are the means ± S.E.M (error bars) of triplicate experiments. (d) Immunohistochemistry staining for pSTAT3 in these tumors demonstrated a decrease in pSTAT3 expression in those treated by MNS1-Leu compared to those treated by vehicle.

To validate the anti-tumor effects *in vivo*, we tested MNS1-Leu in a pediatric HGG PDX model (DIPG XVII, H3K27M). Tumor bearing mice were treated by oral gavage for 21 days with increasing concentrations of MNS1-Leu and tumor volume and bioluminescence increased in vehicle treated animals while tumors in drug treated animals did not (**Fig 5A–5C**). Validation of on-target drug effects were observed in tumors by immunohistochemistry (IHC) analysis of pSTAT3 (Y705) in drug treated mice (decreased pSTAT3) compared to control (**Fig 5D**). During 21 days of increasing drug treatment, there was no significant change in body weight (**S2 Fig**). After 21 days, the animals were sacrificed and heart, liver, spleen and kidney were harvested for histological analysis. No signs of acute toxicity were noted while dosing mice with MNS1-Leu, and histological analysis of major organs did not show any pathological findings (**S2 Fig**). Finally, MNS-1 compounds (25 μM) were placed in human plasma at 37°C for up to 96 h and did not show any degradation by mass spectroscopy (**S3 Fig**).

## Discussion

There is strong evidence that STAT3 signaling plays an important role in cancer and more specifically, in HGG tumorigenesis. Although numerous STAT3 inhibitors have been developed, none have made it through clinical trials to date due to various shortcomings ranging from lack of specificity or potency, to off-target adverse effects, or toxicity [31]. Therefore, the development of effective STAT3 inhibitors could prove to be an important strategy for the treatment of both pediatric and adult HGG. We developed a series of STAT3 pathway inhibitors to address this gap. In the present study, we found that our pyrazole-based inhibitors effectively inhibit the STAT3 activation at the level of STAT3 phosphorylation and inhibit translocation to the nucleus. In both adult GBM and pHGG, they successfully downregulated downstream effectors of STAT3, induced apoptosis, prevented proliferation and inhibited cell migration. Preliminary *in vivo* work yielded promising results for translational safety. Overall our results confirm the importance of the STAT3 pathway in HGGs and potentially hold promise for future treatment.

Numerous studies have reported increased STAT3 phosphorylation in GBM, however, detection of STAT3 phosphorylation in these studies range from 9–83% [32, 33] where some show constitutive activation, and others show IL-6 inducible activation. Furthermore, phosphorylation of STAT3 has been found to be an independent prognostic indicator for poor clinical outcome these patients[34]. A number of studies have characterized STAT3 signaling in GBM cells and evaluated the effects of STAT3 inhibition in HGGs [35–42]. Several small molecule inhibitors of STAT3 function, e.g. AG490 [37], AZD1480 [40], LLL12 [36] and WP1066 [35, 42], have demonstrated good antitumor activity in HGG cell lines with low micromolar activities. However, although AG490 (derivatives), AZD1480, WP1066, and curcumin (a natural product) [39] are primarily upstream STAT3 inhibitors that inhibit JAK2 [43], there are other important mechanisms to activate STAT3 besides JAK2, and so JAK2 inhibition alone may not be sufficient to block all STAT3 activation pathways [44–47]. Furthermore, tyrosine kinases, such as JAK2, activate other proteins other than STATs, and so their inhibition may lead to unwanted side effects. Another molecule, LLL12, which is commercially available, was found to inhibit STAT3 phosphorylation, decrease cell viability and induce apoptosis in GBM cell lines in the low micromolar range, but in practice, the compound readily oxidizes leading to difficulties in synthesis [36].

IL-6 is an anti-inflammatory cytokine that is strongly implicated in tumor progression in numerous cancer types [48–54]. Specifically, IL-6 was previously shown to be required for HGG development in a mouse model [55], and to increase tumor cell invasion and angiogenesis [56]. More recently, IL-6 was shown to induce glioma stem cell (GSC) expansion via STAT3 signaling, as measured by CD133 expression, and implied by neurosphere formation, and enhanced growth of intracranial xenografts [57]. In particular, IL-6 has been implicated in a positive paracrine loop in inducing GSC formation from non-GSCs, and this signaling was blocked by STAT3 inhibitors [26]. Since STAT3 binds to the IL-6 receptor through its SH2 domain’s interaction with a phosphotyrosine sequence (specifically, on the gp130 segment of the IL-6 receptor) [58], we posited that our small molecule inhibitors, with a phosphotyrosine mimetic, could block such interactions, thus preventing its phosphorylation and further activation.

Admittedly, there are other known cytokines that activate STAT3, e.g. IL-4 and -13, and therefore the dependence of our results on IL-6 effect alone is not confirmed as of yet, as it requires validation in other glioma cell lines outside of the ones we tested. In addition, our binding data suggests this is not by disruption at the SH2 domain (Y705) of STAT3, and consequently our compounds inhibit STAT3 phosphorylation by a different mechanism. Our results here show that IL-6 mediated STAT3 phosphorylation is reduced in a dose dependent manner with our inhibitors, and this effect is not likely due to direct JAK2 inhibition. Furthermore, in the presence of our compounds, STAT3 does not translocate to the nucleus in the presence of IL-6, which was confirmed by western blot and confocal microscopy. Ultimately, further investigation is required to determine the exact mechanism of STAT3 inhibition by this compound, and comparison to direct STAT3 inhibitors will prove meritorious in understanding whether or not this mechanistic discrepancy is of any translational significance.

In terms of biological relevance, understanding how these findings can translate into effective tumor treatments for patients is crucial. In our preliminary *in vivo* work, we did not observe any toxic sequelae of MNS1-Leu in a xenograft model despite an obvious decrease in tumor size, which highlights a favorable therapeutic profile. Oral dosing of MNS1-Leu also decreased active STAT3 (pSTAT3) in these tumors, confirming on-target drug effects with oral administration. Additionally, preliminary pharmacokinetic studies show these compounds are quite stable in human plasma.

Looking forward, we anticipate more granular investigation of STAT3 in HGG to evaluate immunocompetent models to account for the possible interplay of multiple cytokines modulating the STAT3 pathway affected by our compounds. Furthermore, assessment of the downstream effects of targeting the STAT3 pathway will shed light onto the role of our compounds in affecting the epithelial-to-mesenchymal transition (EMT) of HGG cells both *in vitro* and *in vivo*, as STAT3-dependent transcription of genes observed in HGGs such as SNAIL1, ZEB1, HIF-1α, and TWIST have been posited to regulate this phenomenon.[59]

## Conclusion

In summary, we have designed a series of pyrazole-based compounds that target the STAT3 pathway. These compounds are potent, dose-dependent inhibitors of STAT3 signaling in HGG cell lines and inhibit tumor cell proliferation via apoptosis. Furthermore, they are selective for tumor cells over normal brain cells, with a good therapeutic window. Oral dosing in a PDX model did not produce any toxic effects and showed a decrease in tumor size and on-target effects in the tumor. Our results highlight the importance of STAT3 signaling in HGG and further support the development of STAT3 inhibitors for their treatment.

## Supporting information

S1 Fig TR-FRET STAT3 binding assay. Evaluation of direct STAT3 SH2 (Y-705) binding of MNS1 compounds by TR-FRET. The fluorescent direct (SH2) STAT3 peptide (5-FAM–G(pY) LPQTVCONH2) was incubated with STAT3 and varying concentrations of inhibitors including MNS1-Leu, MNS1-MV, S31-1757 (a STAT3 inhibitor with published SH2 binding) [30], and STA-21 (parent compound of LLL12) a STAT3 inhibitor developed by virtual screening of compounds predicted to be direct SH2 inhibitors [15]. TR-FRET analysis shows only S3I-1757 (IC_50_ 62.1 ± 6.1 μM) had direct SH2 binding, while MNS1 compounds and STA-21 did not.
(EPS)

S2 Fig Biological monitoring and analysis of major organs following 21 days of MNS1-Leu dosing. (a) There was no difference in body weight between control and MNS1-Leu treated animals. Histological analysis of (b) liver and (c) spleen with hematoxylin and eosin did not demonstrate any microscopic toxic signs after MNS1-Leu treatment.
(EPS)

S3 Fig Human plasma stability of MNS1 compounds. Both MNS1-MV and MNS1-Leu were incubated in human plasma at 37°C over 96 h. Compounds were analyzed by LC-ESI-MS at time points 0, 3, 6, 12, 24, 48, and 96 h. There was no degradation during this study.
(EPS)
